# Sleep onset uncovers thalamic abnormalities in patients with idiopathic generalised epilepsy

**DOI:** 10.1016/j.nicl.2017.07.008

**Published:** 2017-07-12

**Authors:** Andrew P. Bagshaw, Joanne R. Hale, Brunno M. Campos, David T. Rollings, Rebecca S. Wilson, Marina K.M. Alvim, Ana Carolina Coan, Fernando Cendes

**Affiliations:** a Centre for Human Brain Health, University of Birmingham, Birmingham, UK; b School of Psychology, University of Birmingham, Birmingham, UK; c Clinical Physics and Bioengineering, University Hospital Coventry and Warwickshire, Coventry, UK; d Neuroimaging Laboratory, Department of Neurology, University of Campinas, Campinas, Brazil; e Department of Neuroscience, Queen Elizabeth Hospital Birmingham, UK

**Keywords:** Functional connectivity, Generalised epilepsy, Sleep, Thalamic reticular nucleus thalamus

## Abstract

The thalamus is crucial for sleep regulation and the pathophysiology of idiopathic generalised epilepsy (IGE), and may serve as the underlying basis for the links between the two. We investigated this using EEG-fMRI and a specific emphasis on the role and functional connectivity (FC) of the thalamus. We defined three types of thalamic FC: thalamocortical, inter-hemispheric thalamic, and intra-hemispheric thalamic. Patients and controls differed in all three measures, and during wakefulness and sleep, indicating disorder-dependent and state-dependent modification of thalamic FC. Inter-hemispheric thalamic FC differed between patients and controls in somatosensory regions during wakefulness, and occipital regions during sleep. Intra-hemispheric thalamic FC was significantly higher in patients than controls following sleep onset, and disorder-dependent alterations to FC were seen in several thalamic regions always involving somatomotor and occipital regions. As interactions between thalamic sub-regions are indirect and mediated by the inhibitory thalamic reticular nucleus (TRN), the results suggest abnormal TRN function in patients with IGE, with a regional distribution which could suggest a link with the thalamocortical networks involved in the generation of alpha rhythms. Intra-thalamic FC could be a more widely applicable marker beyond patients with IGE.

## Highlights

• Sleep onset modifies thalamic FC in generalised epilepsy differently to controls.
• Differences are regionally specific.
• Regions connected to somatomotor/occipital cortices are consistently affected.
• Intra-thalamic FC may be a surrogate marker of thalamic reticular nucleus function.

## 1 Introduction

Idiopathic generalised epilepsy (IGE) represents 15–20% of all epilepsies (Jallon and Latour, 2005). Its most common electrophysiological feature is generalised spike and wave discharges (GSWD, Seneviratne et al., 2012), which are generally considered to arise from abnormal thalamocortical circuitry, with a longstanding debate about the relative importance of the thalamus and the cortex in their initiation and maintenance (Avoli, 2012, Avoli and Gloor, 1982, Kostopoulos, 2000, Leresche et al., 2012, Meeren et al., 2009). The feline penicillin generalised epilepsy model (Avoli and Gloor, 1982, Kostopoulos, 2000) suggested that GSWD represent a pathological use of the circuitry normally responsible for generating sleep spindles (one of the electrographic hallmarks of stage N2 sleep (Iber et al., 2007)). It has also been suggested that the initiating drivers of GSWD lie in somatosensory cortex (Meeren et al., 2002, Meeren et al., 2009), with subsequent large-scale synchronisation and oscillations of corticothalamic loops. While the precise roles of cortex and thalamus remain under debate (Leresche et al., 2012, Pinault and O'Brien, 2005), the role of the thalamus in the pathophysiology of IGE is well established (Avoli, 2012, Beenhakker and Huguenard, 2009).

Human neuroimaging studies have supported the involvement of the thalamus in GSWD (Aghakhani et al., 2004, Gotman et al., 2005, Moeller et al., 2010), with particular activation of the anterior and centromedian parafascicular nuclei (Tyvaert et al., 2009). Independently of the presence of GSWD, alterations in thalamocortical functional connectivity (FC) have also been seen in patients with IGE (Kim et al., 2014, Wang et al., 2014, Zhang et al., 2011), while structural neuroimaging has identified thalamocortical abnormalities in patients with IGE (Barreto Mory et al., 2011, Bernhardt et al., 2009). While these observations suggest a baseline of thalamic abnormality in people with IGE, the network that is activated at the time of GSWD appears not to be inherently abnormal (Moeller et al., 2011), highlighting the transient and paroxysmal nature of the discharges themselves.

Given the importance of the thalamus and thalamocortical networks during sleep (Hobson and Pace-Schott, 2002, McCormick and Bal, 1997, McCormick et al., 2015), and particularly in the generation and maintenance of sleep spindles and K-complexes (Amzica and Steriade, 2002, Beenhakker and Huguenard, 2009, Colrain, 2005), it is perhaps not surprising that there is a clear relationship between sleep and IGE (Bagshaw et al., 2014, Halász, 2012, Halász, 2013, Kotagal and Yardi, 2001). For example, it has been suggested that GSWD occur preferentially at specific phases of the sleep-wake cycle, particularly in light sleep and transitional phases (Drinkenberg et al., 1991, Halász et al., 2002, Lannes et al., 1988), while sleep deprivation is widely used clinically as an activating procedure for GSWD (Seneviratne et al., 2012, Serafini et al., 2013).

To date, there have been no neuroimaging investigations of patients with IGE during sleep. In this study, we used EEG-fMRI to investigate thalamic FC in patients with IGE and healthy control subjects during wakefulness and light sleep (i.e., N1), postulating that sleep would act as an endogenous perturbation of thalamic and thalamocortical networks. We concentrated on periods without GSWD and applied a parcellation of the thalamus based on cortical FC profiles (Hale et al., 2015), which has previously been used to investigate changes in thalamic and thalamocortical FC in the descent into sleep (Hale et al., 2016). We hypothesised that sleep would highlight alterations to thalamic FC in patients with IGE compared to control subjects.

## 2 Materials and methods

### 2.1 Participants and data acquisition

Twenty-one control subjects and 15 patients with a clinical diagnosis of IGE (ten with juvenile myoclonic epilepsy) were scanned with EEG-fMRI at 3 T (Philips Achieva, controls at University of Birmingham, patients at UNICAMP). The control subjects had no personal history of neurological, psychiatric or sleep disorder, and are the same participants who contributed to Hale et al., 2016. Written informed consent was obtained from all participants, and the study was approved by the Research Ethics Board of the University of Birmingham and the Ethics Committee of the University of Campinas.

EEG data were acquired with a 64 channel MR compatible EEG system (BrainProducts, Germany) from 62 Ag/AgCl MR-compatible scalp electrodes positioned according to the international 10–20 system, with an extra channel to record the electrocardiogram (EasyCap, Brain Products, Germany). Electrode impedances were maintained below 20 kΩ, and EEG data acquisition was synchronised with the MR scanner clock (Syncbox, Brain Products, Germany) (Mandelkow et al., 2006, Mullinger et al., 2008). Brain Vision Recorder (Brain Products, Germany) was used at a sampling rate of 5 kHz with hardware filters of 0.016–250 Hz.

fMRI data were acquired using a 32 channel head receive head coil with whole brain EPI (3 × 3 × 4 mm, TR = 2 s, 32 slices, TE = 35 ms, 1250 volumes per scan for controls, 450 volumes per scan for patients to allow additional communication, with the same sequence used at both sites). Respiratory and cardiac fluctuations were recorded using a pneumatic belt around the upper abdomen and a vectorcardiogram or pulse meter. A high resolution (1 mm isotropic) T1-weighted, anatomical scan was also acquired.

Participants were given minimal instruction prior to scanning, but told not to resist sleep. Scanning sessions were terminated either upon the request of the participant or at the end of the available scanner time. Subjects who demonstrated wakefulness and sleep stage N1 were included in the study, leading to a final sample of 15 controls (9 male, 26 ± 4 yrs, range 21–34 yrs) and 10 patients with IGE (3 male, 32 ± 10 yrs, range 15–47 yrs).

There was no significant difference between the ages of the patient and control groups (two-sample *t*-test, equal variances not assumed, t(10.5) = 1.71, p = 0.12), or between the mean relative displacement of the two data sets (0.09 ± 0.04 mm for controls, 0.10 ± 0.04 mm for patients, two-sample *t*-test, equal variances not assumed, t(23) = 0.94, p = 0.36). Clinical and demographic details of the included patients are shown in Table 1.

**Table 1:** Brief clinical and demographic information for the included patients.

Patient	Age (y)	Gender	Diagnosis	First seizure (y)	Seizure types			AED (mg/day)
					GTCS	Absences	Myoclonia	
1	41	F	JME	10	Yes	Yes	Yes	VPA 250, CLN 1.25, CBZ 800
2	41	F	JME	13	Yes	No	Yes	LTG 200
3	39	F	JME	21	Yes	No	Yes	VPA 750, LTG 200, CLN 6
4	15	F	JME	14	Yes	Yes	Yes	VPA 500, LTG 200
5	32	F	JME	14	Yes	No	Yes	VPA 500
6	21	M	JME	1	Yes	No	Yes	VPA 500
7	23	M	JME	19	Yes	No	Yes	VPA 500
8	30	M	JME	11	Yes	No	Yes	VPA 100
9	47	F	JME	6	Yes	Yes	Yes	LTG 300
10	26	F	JAE	7	Yes	Yes	No	VPA 750, CLN 4

Abbreviations: GTCS – generalised tonic-clonic seizures, F – female, M – male, JME – juvenile myoclonic epilepsy, JAE – Juvenile Absence Epilepsy, VPA – valproic acid, CLN – Clonazepam, CBZ – Carbemazepine, LTG – Lamotrigine, TPM – Topiramate.

### 2.2 Data processing

Gradient and ballistocardiogram artefacts were removed from the EEG data using BrainVision Analyzer 2 (BrainProducts, Germany) and EEG data sleep staged in non-overlapping 30 s epochs by an experienced neurophysiologist according to guidelines from the American Association of Sleep Medicine (Iber et al., 2007). fMRI data were preprocessed using the same methodology as reported previously (Hale et al., 2015, Hale et al., 2016). Briefly, the effects of physiological noise were reduced using RETROICOR (Glover et al., 2000), white matter, CSF, motion parameters and the whole brain signal were regressed out. Five cortical regions of interest (ROIs) covering occipital-parietal, motor, somatosensory, temporal and prefrontal cortices were defined by combining masks from the Harvard-Oxford cortical atlas (http://fsl.fmrib.ox.ac.uk/fsl/fslwiki/Atlases, Hale et al., 2016). The thalamus ROI was defined from the Oxford thalamic connectivity atlas (Behrens et al., 2003). Sub-regions of the thalamus were identified based on their predominant thalamocortical FC to one of the five cortical regions. This resulted in bilateral thalamic masks representing thalamic regions that were most associated with occipital-parietal (OCC), motor (MOT), somatosensory (SOM), temporal (TEM) and prefrontal (PRE) cortices. These bilateral masks were split into left and right hemispheres, and the average BOLD time series calculated and subsequently split into non-overlapping 30 s epochs. Epochs demonstrating GSWD on EEG were excluded from the analysis.

Three types of FC were calculated: (1) thalamocortical FC between each thalamic sub-region and its primary cortical area, (2) inter-hemispheric thalamic FC between homologous left and right lateralised thalamic sub-regions and (3) intra-thalamic FC calculated between each of the five thalamic sub-regions and averaged across right and left hemispheres. In all cases, Pearson correlation coefficients were converted to z-scores using Fisher's transform with Bartlett correction. These values representing FC in each epoch were averaged according to the EEG sleep staging, yielding average FC for each of the three measures per participant per sleep stage.

### 2.3 Statistical analysis

Statistical analysis was performed using SPSS (version 23, IBM Inc., USA). For each of the three types of FC defined above, a three-way split-plot ANOVA investigated the between-subject factor of participant Group (two levels: controls, patients), and the within-participant factors of sleep stage (factor Stage, two levels: W, N1) and thalamic subdivision (factor Region, five regions for thalamocortical analyses, five paired connections for inter-hemispheric analysis, 10 pairs of connections for intra-thalamic analysis: OCC, MOT, SOM, PRE, TEM). The mean relative displacement per participant was included as a covariate in the model, as was the mean-centred valproic acid (VPA) dose as the most consistent anti-epileptic medication. Further pairwise comparison *t*-tests were conducted to ascertain the direction of significant effects, with a two tailed, Bonferroni corrected significance threshold of p < 0.05. Only significant effects are reported and if Mauchly's test indicated a violation of the assumption of sphericity within any of the models the Epsilon Greenhouse-Geisser correction was reported. Since the age ranges of the patient and control groups were different, as a control we repeated the above analyses excluding the oldest and youngest of the patients to ensure that the effects we observed were not driven by age differences in the two groups.

## 3 Results

### 3.1 Thalamocortical FC

**Fig. 1:**
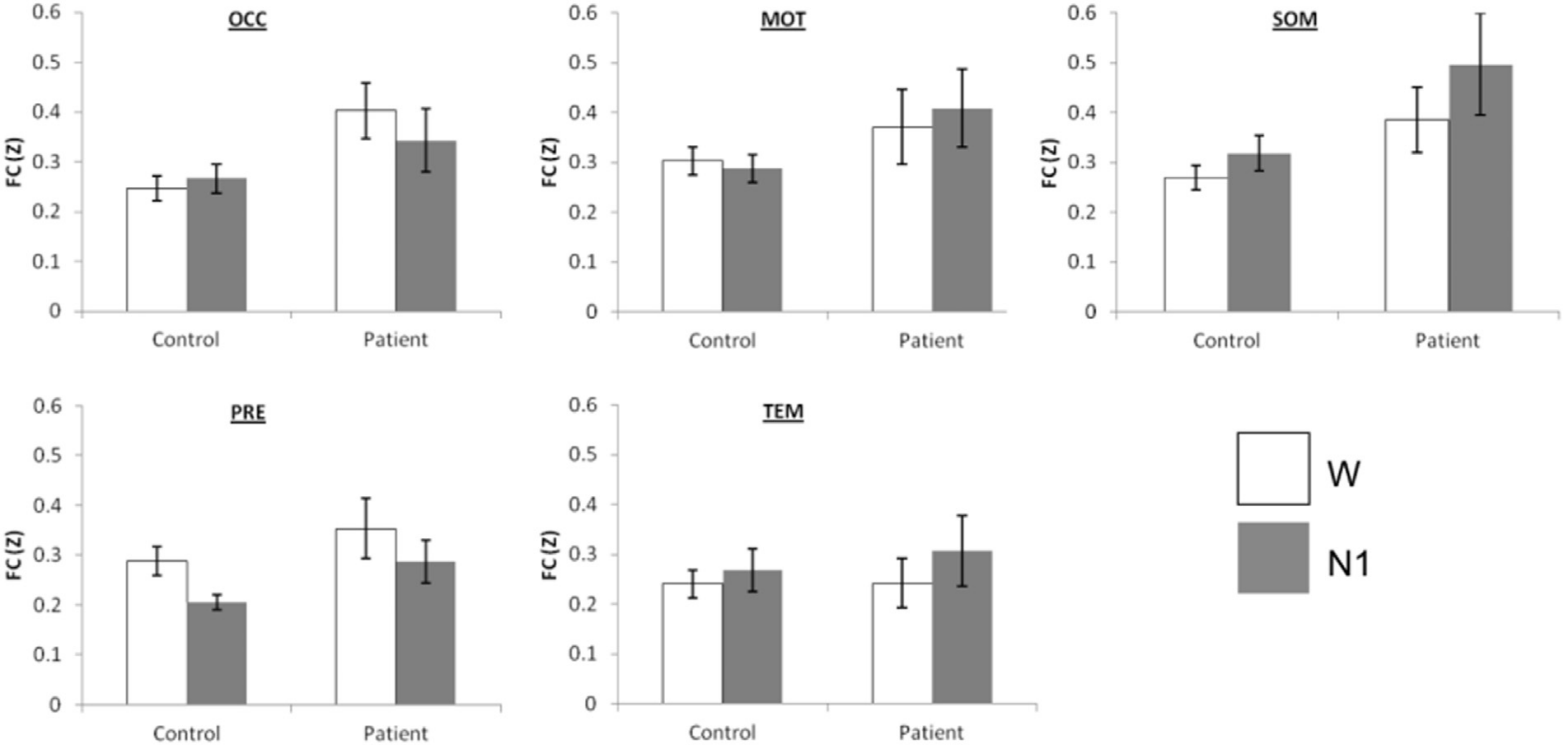
Thalamocortical FC was generally higher in patients with IGE, and the SOM and PRE regions demonstrated a differential response to sleep onset. W: Wake, N1: non-REM sleep stage 1. Error bars represent standard error.

Overall, the thalamocortical FC of patients was significantly higher than controls (Fig. 1, F(1,23) = 8.14, p = 0.009), and there was also a significant Stage ∗ Region interaction (F(4,92) = 2.53, p = 0.047). Further pairwise comparisons indicated a significant differential response to sleep onset in SOM (p = 0.031) and PRE (p = 0.020), with SOM sleep onset indicating an increase in FC, whereas for PRE there was a decrease. Removing the oldest and youngest patients from the analysis resulted in the Stage ∗ Region interaction not achieving statistical significance (p = 0.149). Other effects remained significant.

### 3.2 Inter-hemispheric thalamic FC

**Fig. 2:**
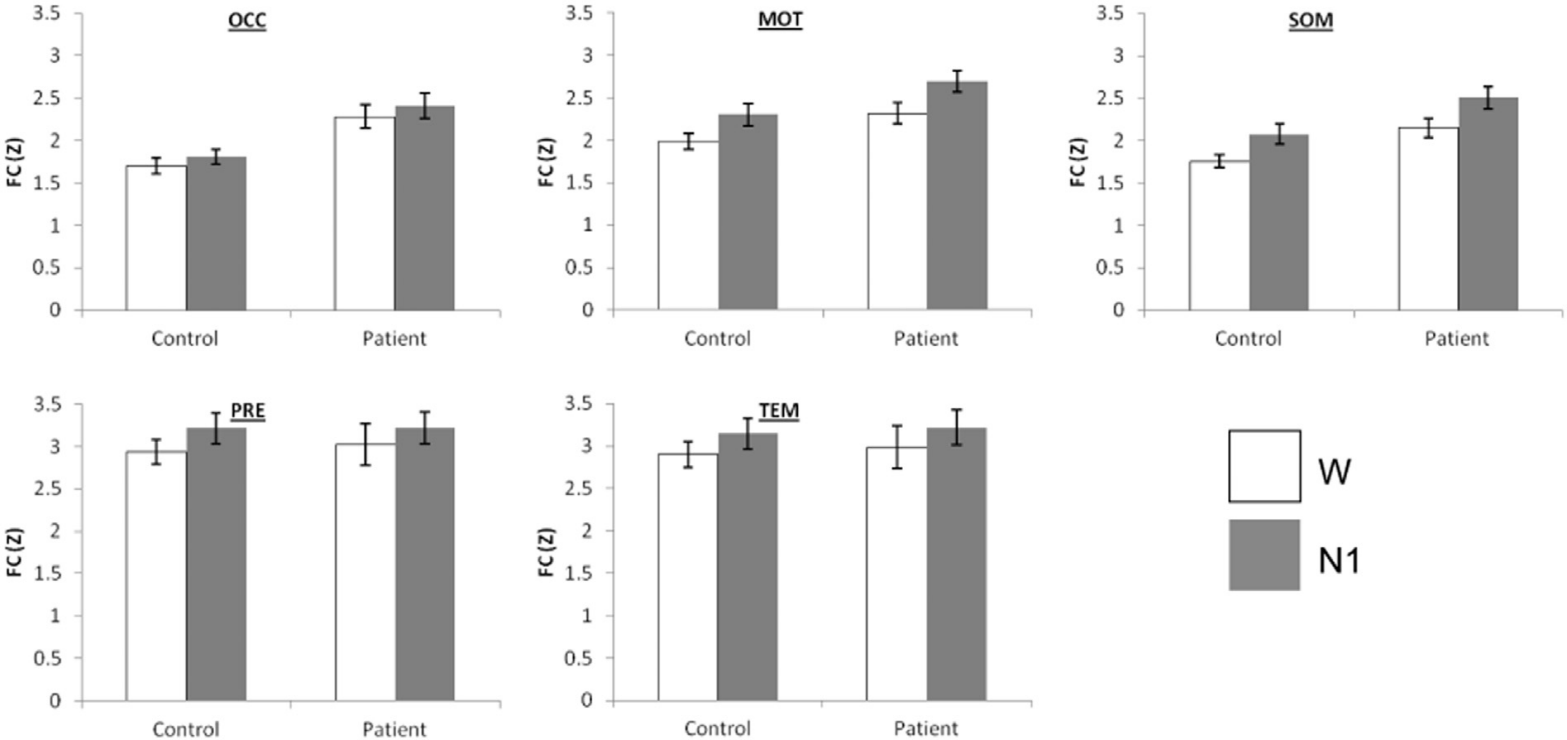
Inter-hemispheric thalamic FC was increased by sleep onset. Patients with IGE had generally increased FC compared to control subjects as well as specifically during sleep in OCC and SOM. W: Wake, N1: non-REM sleep stage 1. Error bars represent standard error.

There was a borderline effect of sleep onset on inter-hemispheric FC (Fig. 2, F(1,23) = 4.29, p = 0.051), with an overall increase in FC. There was also a significant main effect of Region (F(4,92) = 17.83, p < 0.001, Fig. 2). There was a significant three-way interaction of Group ∗ Stage ∗ Region (F(4,92) = 6.63, p = 0.003), with pairwise comparisons indicating differences between patients and controls which were significant in only some of the regions, specifically SOM (W: p = 0.011; N1: p = 0.045) and OCC (N1: p = 0.002), with a borderline effect in MOT (W: p = 0.06). This analysis suggests that the most pronounced differences between patients and controls occurred in wakefulness in SOM, and during N1 in OCC (Fig. 2). These results were largely maintained when removing the oldest and youngest patients from the analysis. The effect of sleep onset was still borderline (p = 0.06). The three-way interaction remained significant (p = 0.002), with the only changes to the pairwise comparisons being on the cases with borderline significance (SOM N1 p = 0.056, MOT W p = 0.04).

### 3.3 Intra-thalamic FC

**Fig. 3:**
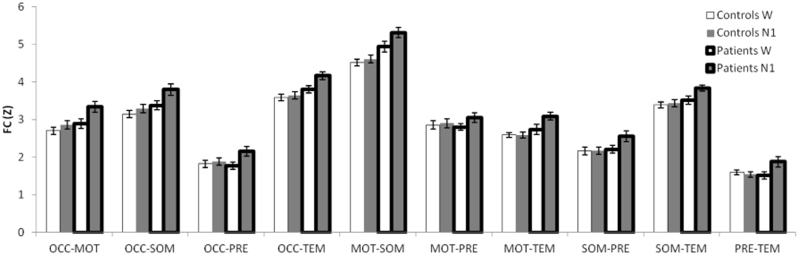
Intra-thalamic FC was higher in patients with IGE than control subjects, due to the increased FC in patients during N1. Patients and controls differed in FC in several pairs of regions that always included either OCC, MOT or SOM (OCC-TEM, MOT-SOM, MOT-TEM). W: Wake, N1: non-REM sleep stage 1. Error bars represent standard error.

Overall, intra-thalamic FC was found to be significantly higher in patients than controls (Fig. 3, F(1,23) = 4.52, p = 0.045). There was also a significant main effect of Region (F(9207) = 94.64, p < 0.001). A Group ∗ Stage interaction was found to be significant (F(1,23) = 9.60, p = 0.005), with pairwise comparisons suggesting that patients had a higher FC than controls during N1 (p = 0.009). The Group ∗ Region interaction was also significant (F(9,23) = 3.97. p = 0.016), with patients and controls differing in their FC for specific pairs of regions (Fig. 3, significant in OCC-TEM p = 0.016, MOT-SOM p = 0.001, MOT-TEM p = 0.012, borderline in OCC-SOM p = 0.053 and SOM-TEM p = 0.049). These results were not greatly affected by removing the oldest and youngest patients from the analysis. The main effect of Group became of borderline significance (p = 0.075), as did the Group ∗ Region interaction (p = 0.055). The borderline paired comparisons in the Group ∗ Region interaction became non-significant (OCC-SOM p = 0.146, SOM-TEM p = 0.12). Other conclusions were not affected.

## 4 Discussion

This study is the first demonstration of alterations to thalamic functional connectivity (FC) during sleep in patients with idiopathic generalised epilepsy (IGE). The thalamus is a crucial structure for the onset and maintenance of sleep and the pathophysiology of IGE (Avoli, 2012, McCormick and Bal, 1997, McCormick et al., 2015, Steriade and LLinás, 1988, Steriade and Timofeev, 2003). As such, it may serve as the underlying basis for the links between sleep and IGE, including sleep onset, stage transitions and deprivation acting as exacerbating factors for seizures and generalised spike-wave discharges (Drinkenberg et al., 1991, Halász et al., 2002, Lannes et al., 1988, Seneviratne et al., 2012, Serafini et al., 2013). Patients and controls differed in all three measures of thalamic FC, and during wakefulness and sleep, indicating disorder-dependent and state-dependent modification of thalamic FC, as well as their interaction.

The most specific differences between patients and controls were observed in intra-thalamic FC, both within and between hemispheres. In control subjects, the onset of sleep did not affect intra-thalamic FC, although it has previously been reported that changes are observed in control subjects in N2 (Hale et al., 2016). Sleep onset lead to an increase in FC which was most consistently observed in thalamic regions that were predominantly functionally connected to somatomotor (SOM, MOT) and occipital (OCC) neocortices. Inter-hemispheric FC differed between patients and controls in SOM during wakefulness, and OCC during sleep. Intra-hemispheric thalamic FC was significantly higher in patients than controls following sleep onset, and disorder-dependent alterations to FC were seen in several thalamic regions always involving either SOM, MOT or OCC.

It is generally considered that there are no direct connections between thalamic nuclei (Kaas and Ebner, 1998), but rather that interactions between thalamic sub-regions are mediated by the thalamic reticular nucleus (TRN), both for intra-thalamic (Crabtree, 1999, Crabtree et al., 1998, Crabtree and Isaac, 2002, Kaas and Ebner, 1998, Lam and Sherman, 2011) and thalamic inter-hemispheric (Raos and Bentivoglio, 1993) communication. It therefore seems likely that this indirect route is driving the alterations to intra-thalamic FC in IGE that we have observed.

The TRN is a purely inhibitory (GABAergic) nucleus which surrounds the main body of the thalamus (Steriade, 2005). It has been implicated in the maintenance of attention (Halassa et al., 2014, Suffczynski et al., 2001, Wimmer et al., 2015) and a diverse range of pathologies from autism-spectrum and attention-deficit disorders (Wells et al., 2016) to schizophrenia (Ferrarelli and Tononi, 2011) and Alzheimer's disease (Hazra et al., 2016). Most pertinently for the current study, TRN-induced inhibition of thalamocortical neurons may underlie the disconnection from the external environment that is observed in sleep and absence seizures, as incoming sensory signals will not be relayed to the neocortex (Steriade, 2005). Alterations to intra-thalamic FC may therefore indicate abnormal inhibitory thalamic control in people with IGE. Since the current data were acquired in the interictal state and excluding epochs with epileptic activity, these abnormalities are present even in the absence of explicit epileptic phenomena, but become apparent following sleep onset.

Changes to intra-thalamic FC with sleep in controls (Hale et al., 2016) and patients with IGE were exclusively increases, with the changes associated with IGE occurring in lighter sleep than in control subjects (i.e., in N1 rather than N2). The precise mechanisms by which alterations of inhibitory control (e.g., via the TRN) might drive changes to FC requires further clarification, but this increase in FC would indicate increased intra-thalamic interaction, and could underlie the link between IGE and sleep. Whether by an initiating discharge in the thalamus or cortex (Lüttjohann et al., 2013, Meeren et al., 2002), increased intra-thalamic interaction would result in a more widespread thalamic response, which presumably would then lead to the global cortical activity that is observed on the scalp EEG.

Despite the relatively coarseness of the thalamic parcellation, regional differences as a function of sleep and between patients and controls were observed. The most consistent changes were in relation to somatomotor and occipital regions, which are characterised by prominent alpha rhythms (8–12 Hz) during wakefulness. The TRN is crucial for the generation and maintenance of cortical alpha rhythms (Suffczynski et al., 2001), the loss of which is one of the primary electrographic features of N1 (Iber et al., 2007). The fact that these regions are affected by sleep and IGE is therefore consistent with the role of the TRN in intra-thalamic FC, and might suggest that alpha-generating thalamocortical networks are involved in driving the alterations that are seen in IGE.

While speculative, this provides an alternative hypothesis to the proposed, and not universally accepted (Leresche et al., 2012), link between GSWD with sleep spindles. There is some support for a link between abnormalities in alpha networks and IGE. For example, increases or decreases to alpha power have been observed prior to GSWD (Inouye et al., 1990), while parental alpha activity has been linked with the observation of GSWD in offspring (Doose et al., 1995), suggesting a genetic association between the two electrographic phenomena. This suggested link therefore requires further investigation.

Although acquired using the same sequences, head coil and the same model of MRI scanner, the patient and control participant data were acquired at two different sites (controls at University of Birmingham, patients at UNICAMP). FMRI data were processed through the same analysis pipeline, which in many cases lead to no FC differences between patients and controls. This is reassuring in relation to the regionally-specific differences which were observed, since it would be expected that any fundamental differences in the image properties would have a more global effect. Physiological noise is often the largest non-neuronal contributor to variance in fMRI data (Murphy et al., 2013), the effect of which can be reduced by RETROICOR as we have done. In addition, the effect of scanner differences on the temporal signal to noise ratio (tSNR), which is an important determinant of BOLD correlations, has been suggested to be small compared to differences between subjects (Huang et al., 2012), leading to FC being reproducible across sites and systems (Jovicich et al., 2016). It therefore seems unlikely that the observed differences between control participants and patients could be driven by the data being acquired at two different sites, but this would need to be confirmed in future studies.

Another obvious difference between the two groups was that the patients were taking anti-epileptic medications, with valproic acid (VPA) being the most consistent across the group. We included the VPA dose in the statistical analysis as a covariate of no interest, and the only available evidence regarding the effect of anti-epileptic medications on FC (Hermans et al., 2015) suggests that anti-epileptic medications lead to a reduction in FC. This is counter to our observations, where increased FC was seen in patients (with one exception of a reduction in prefrontal thalamocortical FC), making it unlikely that medication was driving the differences between patients and controls that we observed. In addition, while the mean age of the control and patient groups did not differ, the patients had a larger age range (21–34 yrs vs 15–47 yrs). While this is not optimal, it seems unlikely to account for the differences between patients and controls that we observed since age-related differences in sleep (Redline et al., 2004) and FC (Andrews-Hanna et al., 2007) tend only to be observed from the sixth decade onwards. This conclusion is supported by the fact that the control analysis whereby the oldest and youngest patients were removed from the data had minimal effect on the results.

During sleep, intra-thalamic FC in patients with IGE differs from control subjects, indicating that sleep onset acted as an intrinsic, internally generated perturbation of the thalamic networks involved in IGE. Interpreting intra-thalamic FC as a surrogate marker of TRN inhibitory control would suggest abnormal TRN function in patients with IGE, the regional distribution of which could be linked with the thalamocortical networks involved in the generation of alpha rhythms. Given the importance of the thalamus and the TRN in a variety of normal and pathological functions, intra-thalamic FC could be a more widely applicable marker beyond patients with IGE.
